# Artificial intelligence adoption in extended HR ecosystems: enablers and barriers. An abductive case research

**DOI:** 10.3389/fpsyg.2023.1339782

**Published:** 2024-01-24

**Authors:** Antarpreet Singh, Jatin Pandey

**Affiliations:** ^1^Organizational Behaviour and Human Resource Management Area, Indian Institute of Management Indore, Indore, India; ^2^Human Resource Area, FORE School of Management, New Delhi, India

**Keywords:** artificial intelligence, optimistic, digital leadership, HR data, partners, AI ethics, human-machine collaboration

## Abstract

Artificial intelligence (AI) has disrupted modern workplaces like never before and has induced digital workstyles. These technological advancements are generating significant interest among HR leaders to embrace AI in human resource management (HRM). Researchers and practitioners are keen to investigate the adoption of AI in HRM and the resultant human–machine collaboration. This study investigates HRM specific factors that enable and inhibit the adoption of AI in extended HR ecosystems and adopts a qualitative case research design with an abductive approach. It studies three well-known Indian companies at different stages of AI adoption in HR functions. This research investigates key enablers such as optimistic and collaborative employees, strong digital leadership, reliable HR data, specialized HR partners, and well-rounded AI ethics. The study also examines barriers to adoption: the inability to have a timely pulse check of employees’ emotions, ineffective collaboration of HR employees with digital experts as well as external HR partners, and not embracing AI ethics. This study contributes to the theory by providing a model for AI adoption and proposes additions to the unified theory of acceptance and use of technology in the context of AI adoption in HR ecosystems. The study also contributes to the best-in-class industry HR practices and digital policy formulation to reimagine workplaces, promote harmonious human–AI collaboration, and make workplaces future-ready in the wake of massive digital disruptions.

## Introduction

Artificial intelligence (AI) has disrupted modern workplaces like never before and has brought massive changes in the way we collaborate, learn, and make decisions (Daugherty and Wilson, 2018; Cortelazzo et al., 2019; Haenlein and Kaplan, 2019; Raisch and Krakowski, 2021). AI not only affects “digital lifestyles” in the workplace (Drubin, 2020, p. 41) but it also impacts the way critical decisions are taken by HR leaders that significantly influences organizational performance.

Artificial intelligence offers many interesting use cases for the human resource management (HRM) domain (Tambe et al., 2019; Garg et al., 2021; Qamar et al., 2021). The field of AI and recent advancements are at the heart of key debates by psychologists and social scientists (Laurent, 2018). While there is strong interest among the research community and practitioners regarding exploring the benefits of AI in HRM, this field is still at a nascent stage and evolving (Basu et al., 2023). Although research in this domain is beginning to accelerate, the AI-HRM academic literature at present is scattered (Qamar et al., 2021, p. 1340). Further, the current literature in the AI-HRM domain lacks a theoretical basis and is patchy and incomplete (Verma et al., 2023, p. 1). This study addresses this lacuna by investigating HRM specific factors that enable and impede AI adoption in extended HR ecosystems and thus aims to plug this significant research gap in the domain of AI adoption in extended human resource management. In this context, this research contributes to the AI-HRM domain by providing a theoretical model for AI adoption in extended HR ecosystems, besides contributing to HRM practice by providing several insights to chief human resource officers (CHROs) that would help them configure best-in-class HR practices at their workplaces.

“Artificial intelligence” (AI) is an umbrella term and refers to a broad class of digital technologies (Krogh, 2018; Haenlein and Kaplan, 2019; Tambe et al., 2019). Haenlein and Kaplan classify AI as analytical, humanized, and human-inspired: pointing to emotional, cognitive, and social aspects at workplaces (p. 6). Haenlein and Kaplan further advocate that “AI will not only impact our lives but fundamentally transform how firms take decisions and interact with employees and customers” (p. 9). AI as a system of algorithms mimics the human brain and performs complex activities such as thinking and decision-making in a similar way as the human brain does (Laurent, 2018; Tambe et al., 2019; Zhao et al., 2022). The algorithms become smarter through training and experiences in exactly the same manner as human beings do (Daugherty and Wilson, 2018; Laurent, 2018). Haenlein and Kaplan (2019) call this iterative process of learning “flexible adaptation” (p. 5).

Human–machine collaboration is a key area for HR professionals as it helps apportion tasks among employees and AI. This phenomenon also entails the selective deskilling of tasks and jobs that were previously in the sole domain of employees. The automation–augmentation dynamics have a profound impact on the new-generation job designs that have the potential to significantly impact organizational performance (Raisch and Krakowski, 2021). A leading enterprise technology corporation, Oracle Corporation (2019), advocates that digital technologies have always been a part of organizational success. Their paper (Oracle Corporation, 2019), further advocates that “the time is now” as regards reimagining ‘human–AI collaboration’ and leveraging the power of AI in HRM. Qamar et al. (2021) also echo a similar sentiment and state that AI offers great promise regarding a “diverse set of use cases in human resource management” (p. 1340). Qamar et al. (2021) cite the lack of adequate research in this domain and express the need for a holistic debate on the current and future impact of AI in HRM (p. 1340).

In recent years, COVID-19 has placed additional demands on HR professionals. As a result, there has been a rapid deployment of digital technology in workplaces that has disrupted existing work practices (Verhoef et al., 2021). HR transformation programs, as part of overall organization wide transformative initiatives, require extensive use of AI. HR managers must lead these change interventions from the front and collaborate with other functional leaders and employees as well as their partners (internal and external). Cortelazzo et al. (2019) refer to this new breed of professionals as e-leaders or digital leaders. Reliable, consistent, unbiased, and trustworthy AI in HRM is the sole responsibility of digital leaders (Laurent, 2018; Cortelazzo et al., 2019). At the same time, a trustworthy AI regulated by an ethical framework is a critical need as AI adoption takes place in the field of HRM. The goal of HR leaders in the context of AI driven transformation is to reimagine workplaces (Daugherty and Wilson, 2018) while ensuring that deskilling is handled in a highly responsible manner, keeping AI ethics centre stage. While there is much debate among the research community and practitioners regarding leveraging AI in HRM, the real picture in industrial workplaces is quite different. There is a stark difference between the hype created by practitioners’ narratives and the reality (Tambe et al., 2019). This requires thorough examination of enablers and barriers to AI adoption in HR ecosystems, both in the context of developing a theoretical model of AI adoption in HRM, as well as the implications for practitioners in terms of embracing best-in-class HR practices with a view to having a well-rounded approach to AI adoption in HRM.

**Figure 1:**
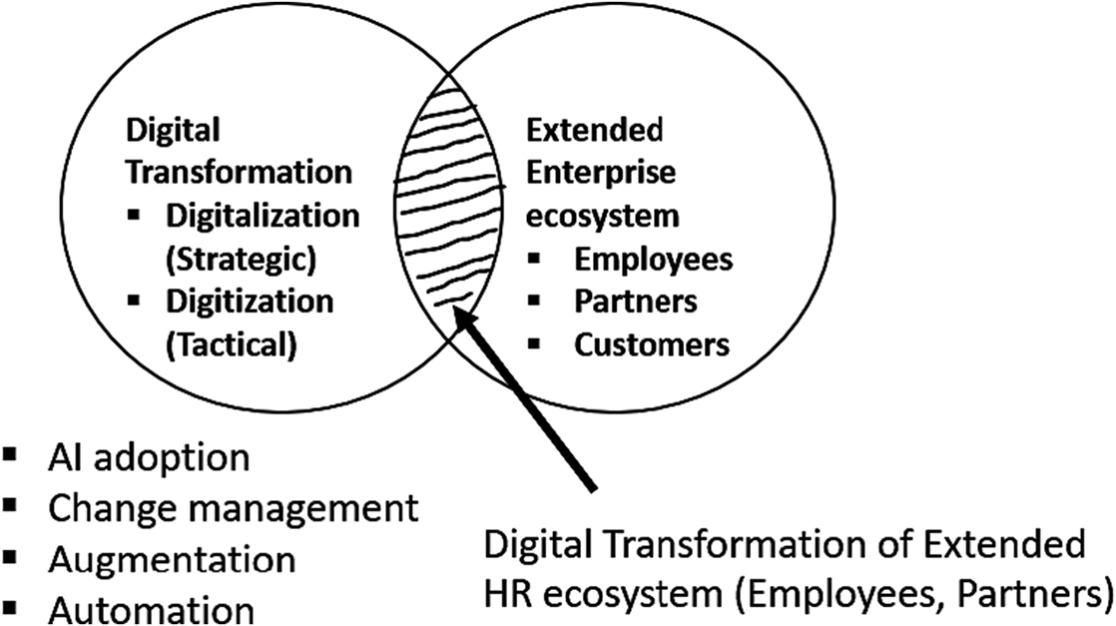
Boundary conditions of the research.

Recent studies on digital transformation have mainly emphasized business strategy and overall organizational outcomes (Trenerry et al., 2021). This study focuses on HRM specific factors and thoroughly investigates the enablers and barriers to AI adoption in extended HR ecosystems. The boundary conditions of this study are shown in Figure 1. The figure highlights the focus of this research that is represented by an area, representing the intersection of digital transformation in an organization and extended HR ecosystems (employees and HR partners).

This research studies three established companies in diverse domains and deploys an abductive case research methodology. This study contributes to the nascent and growing field of AI-HRM academic research by offering a theoretical model of AI adoption in extended HR ecosystems and proposes additions to the unified theory of acceptance and use of technology (UTAUT) framework (Venkatesh et al., 2016). The research findings are also relevant for practitioners, especially chief human resource officers (CHROs) and the HR community, for leading AI-driven initiatives in extended HR ecosystems and delivering superior performance. Digital policymakers at public policy formulation levels also stand to gain in context of digitalizing workplaces and nurturing strategic human capital with a focus on digital upskilling from a macro viewpoint.

### Literature review

Digital transformation in an organization is not merely the adoption of a new set of digital technologies (Kettunen and Laanti, 2017; Trenerry et al., 2021; Verhoef et al., 2021). It is a change initiative that among other things leads to a cultural transformation: where humans and machines co-exist, collaborate, and work harmoniously. In this context of digital technologies adoption, Howcroft and Taylor (2022) advocate that technology is socially shaped (p. 365); thus, technology adoption needs to be viewed in a holistic manner. In the context of human–machine collaboration, several tasks in a job must be judiciously apportioned between humans and machines to enhance organizational performance (Haenlein and Kaplan, 2019; Raisch and Krakowski, 2021). Human intelligence and artificial intelligence must complement each other (Daugherty and Wilson, 2018; Korteling et al., 2021; Raisch and Krakowski, 2021). Humans can reflect on their actions and adapt quickly, whereas algorithms must be trained for adaptation. Human–machine collaboration is a two-way street: the actions of employees shape machine behavior (algorithms are trainable) and algorithms in turn influence employee behavior.

### AI-driven digital transformation

Digital transformation helps ventures grow faster and creates enormous wealth for stakeholders (Khin and Ho, 2019; Proksch et al., 2021; Verhoef et al., 2021). Further, digital transformation led by AI is a continuous change phenomenon that requires a clear vision and a future-ready business model (Zarifis and Cheng, 2022). In this context, digital leadership is key to an organization. In the extended HR ecosystem, it inspires employees and external partners to embrace changes caused by transformative initiatives (Cortelazzo et al., 2019). Digital leadership is seen as a multi-disciplinary concept that besides spearheading technological initiatives brings behavioral changes across an extended ecosystem. The foundation of the digital transformation framework is based on strong digital leadership, harmonious man–machine collaboration, and a digital culture (Khin and Ho, 2019; Proksch et al., 2021; Raisch and Krakowski, 2021). The new generation of digital or e-leaders craft sound digital strategies that lead to harmonious collaboration between humans and machines. Human-machine collaboration refers to the augmentation of digital abilities of humans and machines, as well as the automation of tasks, with machines completely taking it over from humans (Wilkens, 2020; Raisch and Krakowski, 2021). Digital leadership facilitates selective deskilling, with employees giving up tasks that can be performed better and faster using machines. Human employees can thus focus on their core abilities such as feeling, handling emotions, empathizing with customers’ and employees’ issues, and fostering a spirit of collaboration (Haenlein and Kaplan, 2019). Raisch and Krakowski, in the context of AI adoption, encourage managers to embrace the principle of reciprocity. Thus, human employees learn from algorithms and algorithms from employees. The authors term this phenomenon ‘co-evolutionary’ (p. 10).

### Complexities of AI adoption in HRM functions

Recent advancements in digital technologies, including AI, have started transforming HRM ecosystems (Fenech et al., 2019; Garg et al., 2021). Fenech et al. (2019) further point to a massive research gap, in terms of how digitalization at workplaces is experienced by HRM professionals, and state that HRM is a strategic asset of a firm with a focus on enhancing organizational performance (p. 167). Fenech et al. (2019) also discuss the role of digital technologies in transforming HR competencies. Huang et al. (2019) advocate that while AI (with bounded rationality) can provide thinking intelligence, mimicking the human brain for intuitive decision-making, humans can focus on emotions and feelings that AI cannot handle.

AI adoption in HRM is a different ball game to other business functions (Tambe et al., 2019). The authors advocate that decisions related to people can cause serious conflicts within organizations and raise societal concerns. The authors cite an industry use case issue, where a global company had to abort AI-based hiring in 2018 as the algorithms were found to have certain biases that had serious legal and societal implications. Thus, a more humanized approach to the adoption and use of AI in HRM is advisable. Resseguier and Rodrigues (2020) lend support to this argument by stating that things can really go wrong if the potentially harmful impacts of the overuse of technology are not checked (p. 1). The authors state that AI needs to be deployed responsibly so that societal norms are duly respected. The emergence of ChatGPT models has further aggravated the situation regarding the negative impact of AI on jobs (Stahl and Eke, 2023). This places the responsibility of ethically deploying AI squarely on the shoulders of top leaders, including CHROs.

Tambe et al. (2019) further highlight the complexities of using AI in the field of HRM as HR outcomes are quite complex and need managerial judgments. Several HR decisions have serious consequences for employees and society. The authors advise HR managers to collaborate with AI but at the same time use discretion regarding using the results provided by AI (p. 21). This also brings about ethical issues, as AI is not an entity that can be trusted for the simple reason that it does not possess any emotive capabilities (Ryan, 2020). Therefore, AI cannot be held responsible for the HRM outcomes. Hagendorff (2020) cautions about responsible use of AI and warns of a ‘jobless future’ if jobs are deskilled indiscriminately. Morley et al. (2021) also have words of caution regarding the deskilling of jobs and ‘de-responsibilizing’ employees (p. 249) while extensively using AI. Humans must stay in the equation when it comes to the application of AI in workplaces (Krogh, 2018; Cortelazzo et al., 2019; Tambe et al., 2019; Trenerry et al., 2021). Rampersad (2020) too advocates that leveraging AI for business transformation is not a technological challenge but also a human issue (p. 68).

The adoption of AI in HR ecosystems is complex and still in its infancy. There are few studies that aim to contribute to the domain of AI-HRM. As mentioned earlier in the introduction, the existing literature in the field of AI-HRM is scattered and patchy. Table 1 highlights some of the key research studies that have been done in the last 5 years in the AI-HRM domain. None of these studies have focused on providing direct evidence related to enablers and barriers (HRM-related factors) regarding AI adoption in HRM. In addition, the areas related to the role of internal partners (digital subject matter experts, who support HR) as well as external HR partners is understudied. This research study combines both the areas (HRM-factors: enablers and barriers and extended HR ecosystems) and aims to plug this important research gap that exists in the AI-HRM domain. Thus, the adoption of AI in extended HR ecosystems requires intensive examination and validation (through direct evidence) of the HRM-specific factors that enable or impede AI adoption.

**Table 1:** Recent research studies in the AI-HRM domain.

S. No.	Literature reference	Type of research	Theoretical contribution of the research
1	Fenech et al. (2019)	Qualitative research	Digital transformation (changing role of HRM)
2	Raisch and Krakowski (2021)	Literature and book review	Augmented intelligence (automation and augmentation and human–machine collaboration)
3	Tambe et al. (2019)	Conceptual analysis	AI and HRM (opportunities and challenges)
4	Stahl and Eke (2023)	Literature review	AI ethics (ethical issues of emerging technology)
5	Rampersad (2020)	Quantitative analysis	Fears of deskilling (HR automation and deskilling)
6	Cortelazzo et al. (2019)	Literature review	E-leadership (role of digital leadership in the AI age)
7	This research study	Abductive case research	To identify HRM-related factors that enable and impede the adoption of AI in extended HR ecosystems

A review of recent research articles indicates a huge gap between the narrative and reality related to AI adoption in HRM (Tambe et al., 2019). Tambe et al. cite a report from 2018 from the networking platform ‘LinkedIn’ which mentions that only 22% of HR managers have implemented analytics in HR (p. 16). The scale at which AI adoption is happening in the domain of HRM needs to be thoroughly researched. There has been some acceleration in AI adoption in HRM in the last few years, especially due to COVID-19 inducing digital transformation in enterprises (Drubin, 2020; Trenerry et al., 2021; Rozman et al., 2022). At the same time, there is a significant research gap in identifying and examining HRM-specific factors (enablers and barriers) regarding AI adoption in HRM. In addition, several myths regarding the use of AI in HRM have not been comprehensively researched. Common myths include the misconception that AI can create anything, that it will completely replace people, and that it is always neutral. Laurent (2018) advocates that debunking AI myths is an issue of strategic relevance. This would help the HR community look toward artificial intelligence with hope and not fear.

The research gap is addressed effectively by asking the pointed research questions. Dodgson (2020) emphasizes that the “right question is one that needs answering, thus adding to our knowledge base” (p. 105). Dodgson further states that no matter how rigorous a research methodology is, a sound study can only be designed if the research questions are sharp, clear, and easily understood by others. This research aims to plug the research gap as identified in the previous section by asking two pointed research questions related to enablers and barriers, respectively.

Research Question 1 (RQ1): *“What are the human resource management (HRM)-specific factors that enable the adoption of artificial intelligence (AI) in extended HR ecosystems in organizations?”*

Research Question 2 (RQ2): *“What are the human resource management (HRM)-specific factors that inhibit the adoption of artificial intelligence (AI) in extended HR ecosystems in organizations?”*

## Research methodology

Theory building from case research helps examine complexities and novel phenomena (Eisenhardt and Graebner, 2007; Krogh, 2018). Krogh (2018) states that AI is a new and poorly understood phenomena (p. 408) and offers tremendous opportunities for phenomenon-based theorizing and abductive reasoning (p. 405). Bansal et al. (2018) recommend a qualitative approach for research areas that have been “understudied” (p. 1191) empirically and for examining the challenges that are quite unique.

Eisenhardt and Graebner (2007, p. 25) strongly support a research strategy that involves using one or more cases to create theoretical constructs and propositions. Eisenhardt and Graebner also state that theory building from multiple cases yields more robust, generalizable, and testable theory than a single case research (p. 27). Yin (1981) states that case studies are based on a variety of data sources and are rich empirical descriptions of instances of a phenomenon. Extending this argument, Eisenhardt and Graebner (2007) advocate that case studies represent a real-world context in which various phenomena occur. The authors support the case study method stating that such research produces a theory that is accurate, interesting, and testable (p. 26). Eisenhardt and Graebner (2007) further state that the research question is better addressed by building a theory, as these are novel phenomena that are understudied. Therefore, direct theory testing is not beneficial. Eisenhardt and Graebner (2007) also recommend deploying a case study method and stating research question (s) with a broader scope, which will help research to be more flexible.

### Abductive case research

Dubois and Gadde (2002) strongly support case study research by saying that the interaction between a phenomenon and its context can only be clearly understood through case research (p. 554). The authors state that analyzing interdependencies is the key to the research investigating dynamic phenomena. Dubois and Gadde (2002) further state that an abductive approach to case research has a characteristic feature of ‘systematic combining’: it is a process where theoretical framework, fieldwork for collecting data, and case analysis progress concurrently (p. 554). In systematic combining, the theory is confronted with the empirical world and this process of confrontation continues throughout the research (p. 555). The goal of systematic combining, as Dubois and Gadde (2002) state, is to match theory and reality. The authors also point out that the “abductive approach to case research” has the potential to yield more than inductive theorizing and that continuous evolution of a case during the research becomes both a tool and product. This argument is supported by Krogh (2018), who says that the AI decision-making phenomenon is quite suitable for “abductive reasoning” (p. 406). Miller and Brewer (2003) refer to abduction as a creative inspiration that helps a researcher to relate hypothetical explanations with the reality or an empirical fact (p. 2). The authors further state that abduction is an iterative process leading to a single hypothetical explanation that fits well with reality (the empirical world).

This study deploys an abductive case research approach that is well suited to the research question. The adoption of AI in extended HR ecosystems and changing HR landscape must be examined thoroughly from the lens of a human resource professional. This study examines various interdependent processes and phenomena using an abductive case research by investigating AI adoption in the extended HR ecosystems of three business organizations (selected from a diverse set of 12 companies in India by applying a robust selection criteria). The identities of the companies have been masked and only alphabetic codes represent them, as below:

- TS: A well-known brand that designs and delivers software solutions for global clients.
- EC: A leading e-commerce aggregator in the country.
- FG: A fast growing premier fast moving consumer goods (FMCG) brand.

**Figure 2:**
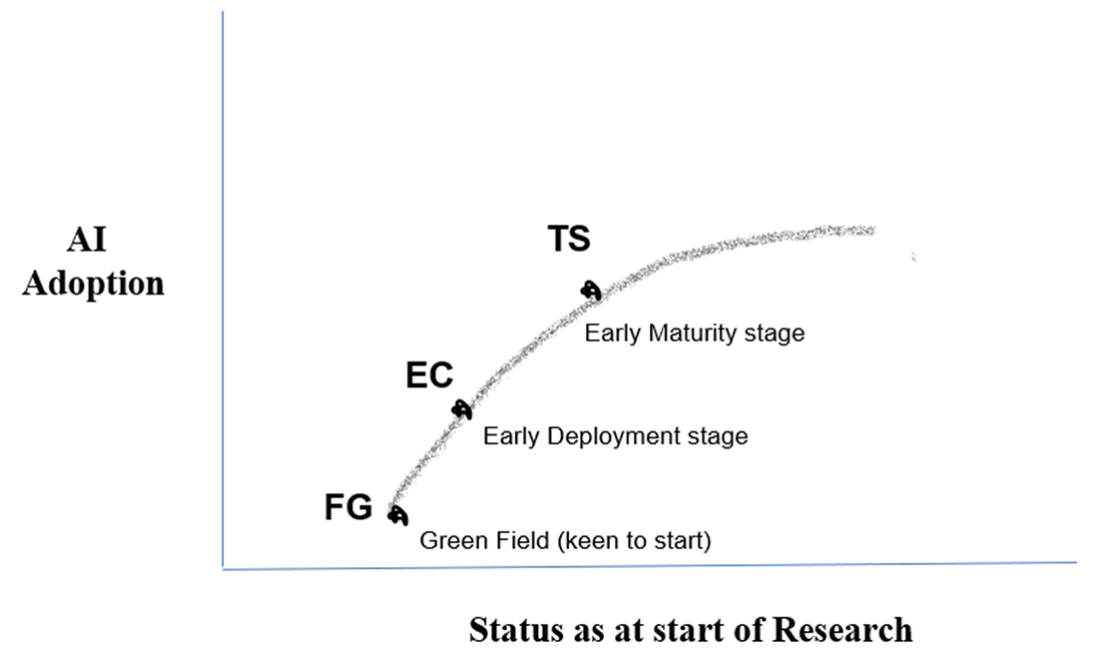
AI adoption levels.

TS and EC have an inhouse capability to develop AI solutions through a team of digital experts who support their HR (internal partner). Both companies also deal with external HR partners. FG is at the greenfield stage. These three companies represent Indian industry as a whole fairly well in terms of scale, business model, employees in HR departments, and relationships with external HR partners. While EC is one of the largest e-commerce companies in the country with a new-age business model, TS represents the software industry quite well. In addition, FG represents the traditional fast moving consumer goods (FMCG) sector, using a brick-and-mortar business model. The three companies are at different stages of AI adoption as shown in Figure 2.

### Sample size, data sources, and coding methodology

The sample size for the study was 27 interviewees including HR employees (chief human resources officers (CHROs) and HR mangers/executives) and digital subject matter experts (SMEs) who support HR as an internal customer for AI adoption. We followed the principle of data saturation to decide when to stop the data collection. Fusch and Ness (2015) advocate that data saturation is not about the numbers but about the depth of the data (p. 1409). This principle has been an integral part of the research design, so that interview questions that yield similar results from different interviewees are not repeated. While deploying a case research design, data must be collected from multiple sources (Ridder, 2017). This study collected data from the following sources:

- Interviews of CHROs (chief human resources officers), HR executives, HRBPs (HR business partners), and digital SMEs (subject matter experts)
- Working group discussions (WGDs) set up by the three organizations related to different areas (six work groups: three at TS, two at EC, and one at FG)
- Secondary sources: company web sites and documents shared by HR managers

In-depth interviews with open-ended questions are a good way to capture interviewees’ feelings and perspectives (Guion et al., 2011). Guion et al. (2011) further recommend that the questions should be structured such that the response of the interviewee should have adequate details. Open-ended questions help respondents to think deeply and share their perspectives. Guion et al. (2011) suggest a seven-step approach to the entire process of “thematizing”, “designing”, “interviewing”, “transcribing”, “analysing”, “verifying”, and “reporting”. The first step, thematizing, relates to the purpose of an interview. The key responsibility of an interviewer is to carefully listen to interviewees and gather all the information. The next step relates to the design that specifies the way information will be collected. Guion et al. (2011) state that step three relates to interviewing and the fourth step relates to transcribing the recorded interviews. The analysis phase entails identifying codes that yield few themes. The sixth step relates to verification or in other words ensuring that the findings are credible. The last step as per Guion et al. (2011) relates to reporting the findings that not only define the work done by the study but also show future directions of the research. Boyce and Neale (2006) stress upon the need to conduct in-depth interviews and further state that in-depth interviews are useful when new issues are to be investigated or explored, thus proving to be appropriate for the current study.

**Figure 3:**
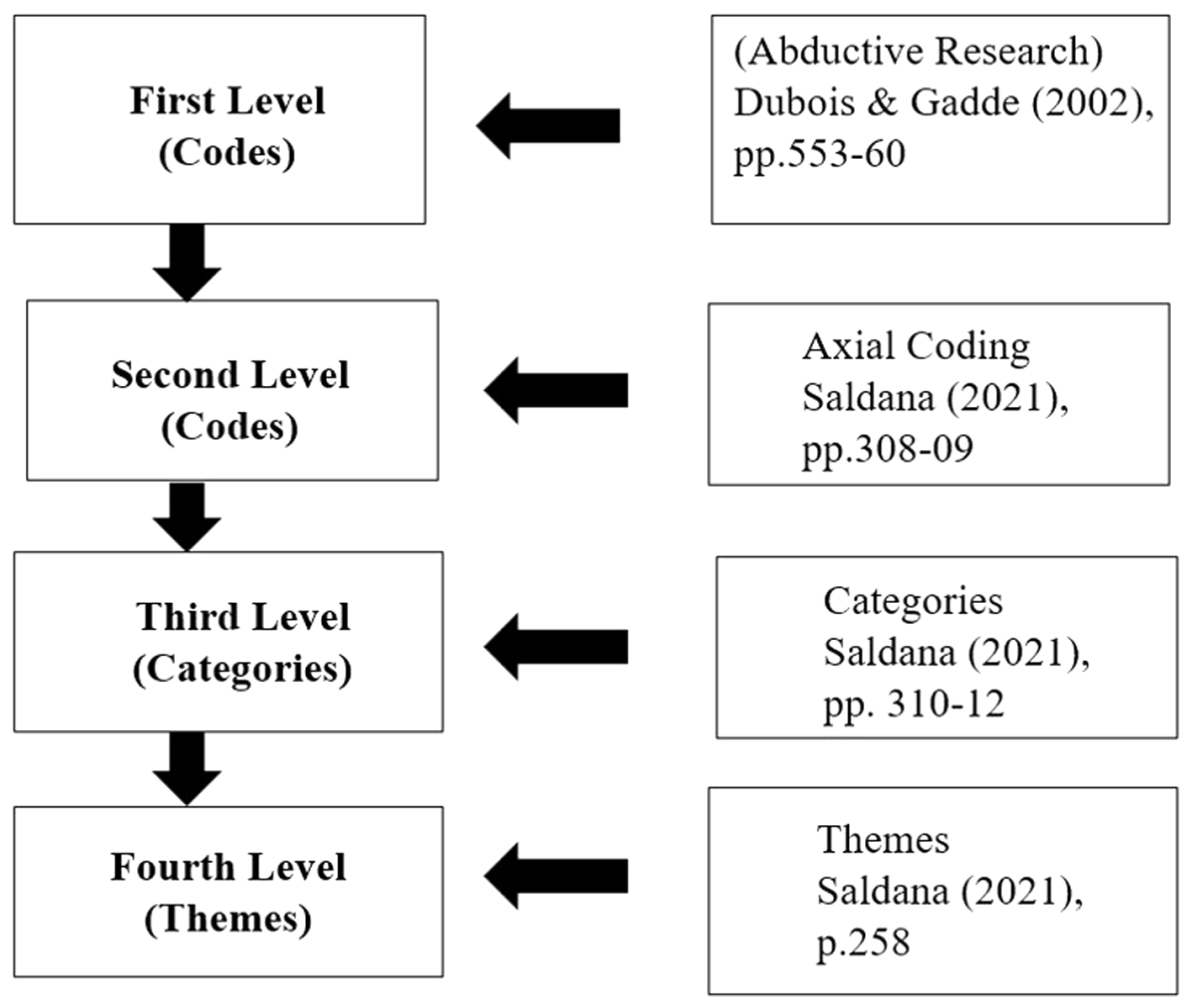
Coding methodology.

Next, the coding methodology as part of the research design includes a step-by-step approach, grounded in the coding methods provided in the literature, as shown in Figure 3. Successive refinements and analysis will help reduce the first level codes to a few categories that further lead to key themes which must help answer the research question (Saldana, 2021, p. 258).

### Trustworthiness

The validity of qualitative research is a challenging field (Creswell and Miller, 2000). Cresswell and Miller further state that qualitative researchers must demonstrate that their studies are ‘credible’ (p. 124). Dubois and Gadde (2002) advocate that combining various sources of data denotes triangulation and helps establish credibility. Dubois and Gadde further state that triangulation not only helps in the accuracy of collected data, but also facilitates the discovery of new dimensions of the research problem (p. 556). Fusch and Ness (2015) emphasize on the need to collect and analyze data from multiple sources. Fusch and Ness (2015) further point out that “triangulation through multiple sources of data will go a long way towards enhancing the reliability of results” (p. 1411). For triangulation, the research compares working group discussions (WGD) data analysis with the results obtained through analysis of interviewees’ data to check the similarity in results. Connelly (2016) states that the trustworthiness of research is key to the “usefulness and integrity” of research findings and provides five components: credibility (confidence in the study), dependability (data being stable during research), confirmability (consistency in a manner that findings can be repeated), transferability (usefulness of research findings in different settings), and authenticity (selecting appropriate interviewees for the research).

Mouter and Noordegraaf (2012) emphasize coding reliability, stating that the method of compressing words into fewer categories needs to be systematic and reliable (p. 1). Furthermore, as per Mouter and Noordegraaf (2012), the inter-coder reliability coefficient should preferably be higher than 0.9.

## Research findings

The deployment of research methodology led to the collection of six sets of WGD data (21 meetings) and interview data (27 interviewees). Johnson and Jehn (2009) advocate that a “case study becomes strong and convincing if findings fit the data set” (p. 124). Johnson and Jehn (2009) further advocate that triangulation deploys a combination of methods to study the same phenomenon (p. 125). The triangulation of results from interviews and working group discussions is highly consistent, truly represent the data set, and have a high level of similarity. This demonstrates the credibility and trustworthiness of this research. In addition, as outlined in the research methodology, the trustworthiness of the research findings has also been established in five factors (Connelly, 2016) which include credibility, dependability, confirmability, transferability, and authenticity.

### Key themes

The results of coding analysis (interviews data) are indicated in Table 2. The five key themes out of a total of 10 themes are discussed below, along with the triangulation of the interview data with the work groups discussions data. This is to help establish the credibility and trustworthiness of the research findings. Comments from interviewees in the context of the five key themes are highlighted (minor moderation has been done in some of the comments to articulate the messages clearly in the context of five key themes).

**Table 2:** Level wise coding analysis and key categories (interview data).

S. No.	Themes	1st	2nd	3rd	Key categories
1	Employees	156	31	17	Optimism, Human touch, Human–machine collaboration, Shared enthusiasm, Whole-hearted support
2	Culture	72	18	10	Culture binds, Digital lifestyles
3	Innovation	42	9	5	Digital innovation, Shared values, Way of life
4	Digital leadership	35	10	6	Role model, Inspirational, People-centric, Technology agnostic
5	HR processes	53	7	5	Digitization, Speed of work, Business alignment
6	Change	72	14	6	Technology-induced anxiety, Trust, Growth pillar
7	HR data	33	6	5	Data quality, Low volume, Data speaks, Robust models
8	Business model	19	4	3	Faster adoption, Non-hierarchical structure
9	Specialized HR partners	10	5	3	Digital enablement, Scale, Leveraging expertise
10	AI ethics	11	3	3	Privacy and security, Ethical guidelines, Public scrutiny
Total	10	503	107	63	

### Employees as enablers and barriers

Optimistic and collaborative employees have emerged as one of the top enablers of AI adoption. One of the three work groups set up by TS is related to employee sentiment analysis. The company uses an AI-based bot to dynamically analyze the sentiments of employees that provides a regular pulse check of employees and helps leaders to provide a human touch and dispel fears of deskilling. A comment from an employee sentiment analysis working group at TS sums it up:

“Employee emotions analysis *tool provides critical inputs for HR leaders*.”

A senior leader from TS talked about the importance of continuous pulse check:

“*Before this AI tool, we used to have an employee engagement survey once a year. The current AI-based method is more like a live, ongoing thing and very targeted.”*

Digital natives across the three companies showed a great deal of optimism in AI adoption. A digital native from a recruitment working group at FG commented:

“*I am very optimistic about AI in HR.*”

Another digital native from EC in their interview sounded highly optimistic:

“*AI is definitely going to, you know, revolutionize the HR* [sic].*”*

Employees have technology-induced anxiety with a fear that AI will make them lose control over their jobs and could eventually replace them. The inability to have a timely pulse check could severely impede the adoption of AI in HR ecosystems. A senior leader from TS comments regarding fear and anxiety among employees because of use of AI technology:

*“I think as we started using AI, those apprehensions went away, people started seeing the results, people started seeing the value that AI brings in.”*

In addition to technology-induced anxiety, a lack of collaboration among employees in HR and digital SMEs (supporting HR as an internal customer) can also prove to be a major barrier for AI adoption in the HR ecosystem of an organization.

Thus, a lack of collaboration with digital SMEs hurts the AI adoption process. The HR leaders must address the challenge of a lack of collaboration among HR employees and digital SMEs to facilitate smooth AI adoption.

### Digital leadership as an enabler

Digital or e-leaders in today’s AI age have both a people as well as a technology orientation. They have open mindsets and help teams embrace digital technologies to create more value. A digital native in the FG recruitment working group commented on leaders with conventional mindsets and viewed it as a barrier to AI adoption:

“*Leaders with conventional mindsets are not very keen in* [sic] *embracing AI in HR.*”

A senior HR leader in TS that successfully adopted AI countered it and highlighted the importance of digital leadership:

“*Leadership being encouraging and supporting you is a big enabler.*”

A digital native from TS commented about digital leadership:

“*Digital leadership is how much the leadership is willing or open to exploring the strength of technology in achieving our goals.*”

Strong and inspirational digital leadership, where leaders walk the talk and embrace AI, is key to the effective adoption of AI in HR ecosystems.

### HR data as enabler and barrier

The results of this study indicate that HR data are one of the key enablers of AI adoption. Well-structured and reliable data are key to providing meaningful insights to HR leaders. HR teams need data that are reliable, structured, and accurate about ground realities. A comment from a TS working group member (learning and development) in the context of the quality of HR data was:

“*HR data set needs* [sic] *to be improved for AI models to work on it.*”

Poor quality of HR data (unreliable, unstructured, etc.) will be a major barrier to AI adoption. In addition to reliability and structurally formatted HR data, the data volume also plays an important role. A digital SME from TS, who supports the HR department (in TS), emphasized the need to have a sufficient volume of HR data for AI adoption and commented:

“*With very less HR data* [sic], *it is difficult to engage good AI models and get good accuracy.*”

Therefore, HR leaders need to ensure that HR data are not only reliable and structured but that there is a reasonable volume for training AI models and obtaining the desired results accuracy.

### Specialized HR partners as enablers

In the context of this research, the HR ecosystem includes internal partners (digital SMEs) and external HR partners providing specialized services for various HR verticals of the organization. TS has taken strategic initiatives to adopt AI in HR and has been an early mover in the industry. Although TS has a strong team of digital SMEs supporting the HR department, the leadership in TS has been proactive in working with external digital partners whenever specialized digital expertise from outside the organization is required (e.g., employee sentiment analysis). The CHRO of TS commented regarding the context of an extended HR ecosystem and leveraging external partners’ expertise wherever required:

“*Bringing in these tools from experts in the market, right!* [sic]*”*

A talent acquisition manager from TS echoed a similar sentiment regarding leveraging partners’ expertise:

“*There are small organizations which you know have specialties in terms of working on certain kinds of technologies.*”

The HR analytics manager from EC also highlighted the need to have external HR partners with the comment:

“*We need digital partners for designing a machine learning-based recruitment solution.*”

In addition, external partners also need to leverage technology as these organizations (small and mid-sized) work with the HR departments of relatively larger organizations. The HR analytics manager of EC in this context commented:

*“We do have certain hiring partners and they like, usually come up with certain trends and certain patterns that they have identified from the data.”*

Partners (internal digital SMEs as well as external HR partners) thus play a significant role in the adoption of AI in extended HR ecosystems. In addition, external HR partners must be digitally enabled to leverage AI in their respective ecosystems, as it will help them to serve their customers (the HR departments of the organizations they are serving) in an effective manner.

### AI ethics as an enabler and a barrier

The growing adoption of AI in business organizations including HR ecosystems has added enormous responsibility to the shoulders of HR leaders to be inclusive and transparent. Deskilling fears must be addressed through a well-rounded set of ethical guidelines. A member of the recruitment working group (FG) commented:

“*Employees fear deskilling of jobs.*”

A digital native from EC spoke of public scrutiny to ensure that the guidelines are openly available in the public domain:

“*We have a sunshine test. As long as AI ethics stands* [sic] *public scrutiny, you can implement it.*”

A digital native also spoke in the context of employees’ privacy and cyber security, saying:

*“Privacy, confidentiality, security, all these aspects, you know, continuously need to be reviewed.”*

A digital native from EC sounded optimistic in terms of organizations embracing AI ethics guidelines:

“*As AI advances, I am sure, proper ethical guidelines will come into place.*”

Thus, the onus is on Chief Executive Officers (CEOs) and CHROs to ensure that employees’ privacy is respected and sufficient safeguards are provided for cyber security that includes protecting employees’ data. In addition, AI ethics guidelines need to be comprehensive and voluntarily embraced by organizations. The lack of focus on well-rounded AI ethics or a failure to embrace AI ethics can prove to be a major barrier to the successful adoption of AI in HR ecosystems.

### Cross-case analysis

Miles and Huberman (1994) stress upon the need to display data effectively in qualitative research. The cross-case analysis in Table 3 presents the big picture and helps to understand how the three companies are leveraging AI for a superior HRM performance. The cross-company matrix indicates scores based on average code frequency (Strong: a score greater than or equal to 2.0, Moderate: 1.0 to 2.0, and Weak: 0). The study also presents a case analysis matrix for digital natives and digital immigrants. Thomas (2011), in the book “Deconstructing Digital Natives”, characterizes digital natives as those who have grown up in the times where digital technologies including the worldwide web (www) have become part of everyday life. Digital immigrants on the other hand have grown up at earlier times and may have different thought patterns and workstyles when compared to digital natives. The case analysis matrix for digital natives and digital immigrants is presented in Table 4.

**Table 3:** Cross-case analysis matrix.

	Theme	Company		
		TS	EC	FG
1	Employees	Strong (3.0)	Strong (2.3)	Strong (2.7)
2	Culture	Strong (2.5)	Strong (2.9)	Strong (3.8)
3	Innovation	Strong (3.3)	Strong (2.2)	Moderate (1.5)
4	Digital leadership	Strong (2.1)	Strong (2.8)	Strong (2.0)
5	HR processes	Strong (4.0)	Strong (2.4)	Strong (4.3)
6	Change	Strong (2.3)	Moderate (1.5)	Strong (2.0)
7	HR data	Strong (2.8)	Moderate (1.2)	Moderate (1.0)
8	Business model	Strong (2.5)	Moderate (1.6)	Moderate (1.0)
9	Specialized HR partners	Moderate (1.4)	Moderate (1.5)	Weak (0.0)
10	AI ethics	Moderate (1.0)	Moderate (1.8)	Weak (0.0)

**Table 4:** Digital immigrants–digital natives case analysis matrix.

S. No.	Theme	Digital natives	Digital immigrants
1	Employees	Strong (2.3)	Strong (4.0)
2	Culture	Strong (2.9)	Strong (3.2)
3	Innovation	Strong (2.2)	Strong (3.3)
4	Digital leadership	Strong (2.2)	Strong (2.6)
5	HR processes	Strong (2.9)	Strong (4.8)
6	Change	Moderate (1.5)	Strong (4.5)
7	HR data	Moderate (1.6)	Strong (2.5)
8	Business model	Strong (2.0)	Moderate (1.0)
9	Specialized HR partners	Moderate (1.6)	Moderate (1.0)
10	AI ethics	Moderate (1.6)	Weak (0.0)

## Discussion

The fast-changing HR landscape is seeing technology play a stellar role in making HR more effective and efficient. Globally, CHROs in major corporations are beginning to have a seat at the table and have enormous responsibility to make their organizations future-ready. In this context, Anderson (2020) quotes “Artificial Intelligence is a superhero in disguise”. Anderson (2020) says that AI is helping HR leaders to make a business impact and be an equal stakeholder in making business successful, resilient, and future-ready. HR leaders in today’s digital age face multi-pronged challenges. AI adoption is quite complex due to a multi-generational workforce (digital natives and immigrants have different thoughts regarding AI). In addition, HR data is intertwined with several qualitative factors (emotions, career aspirations, attitudes, and behaviors) which makes HR outcomes quite complex (Tambe et al., 2019, p. 17).

Several authors support the view that digital transformation is all about people, not just technology (Kettunen and Laanti, 2017; Cortelazzo et al., 2019; Verhoef et al., 2021). Krogh (2018) also describes AI as a “pervasive phenomenon and not average run of the mill technological innovation” (p. 404). The research findings provide overwhelming support for the role of employees in driving change in the context of AI-led digital transformation, which is not just about technology: employees play a pivotal role in the transformation.

AI adoption often induces anxiety and stress among employees, as they fear the loss of a human touch as well as the loss of control over their jobs. Thus, it is important to capture employee sentiments regularly. This is a good use case of AI adoption in HRM and helps CHROs initiate the required measures for the retention of their human capital. Raisch and Krakowski (2021) advocate that people must augment their abilities with AI and that harmonious human–machine collaboration is the way forward rather than replacing people with AI.

Organizations must be digitally agile to combine digital assets and capabilities to create new ways of doing work (Verhoef et al., 2021; Dhondt et al., 2022). This is possible only if digital leaders or e-leaders in HRM functions are inspirational and have a vision. Digital leaders need to augment AI and not substitute human behavior (Krogh, 2018; Raisch and Krakowski, 2021). Einola and Khoreva (2020) advise business and HR leaders that AI implementation can take longer as humans and AI need to change slowly in tandem (p. 130). Thus, this AI-led transformation will have strategic implications for the organization as it goes through a massive phase of change. The vision of CHROs should include leveraging the power of AI on the one hand and the creativity, ingenuity, and uniqueness of employees on the other hand. The change must be managed well, otherwise it could prove to be a double-edged sword and detrimental to an organization’s long-term prospects, including its future readiness. In the wake of AI adoption, digital leaders must have flexible mindsets that encourage experimentation and eliminate fear of failure. Digital leaders induce a culture of trust: a key to the digitalization of the HR function. Goran et al. (2017) highlight the role of leaders and a culture, arguing that organizations that are risk averse and promote siloed mindsets would be ineffective in today’s fast changing digital age.

There are further complexities in HRM owing to the nature of HR data (Tambe et al., 2019). Fernandez and Gallardo (2020) point to unreliable data as a barrier to the adoption of HR analytics. The authors advocate that analytics is all about “leveraging value from data” (p. 163). The availability of data in HR ecosystems is insufficient: it must qualify as a potent source of value creation by being structured and clean. At the same time, a large amount of business data is created that serves as a key input for an overall strategy formulation (Eriksson et al., 2020, p. 796). The availability of validated “business and HR data” is key to sound decision-making in an organization. Verhoef et al. (2021) talk of a “digitization” phase as integral to digital transformation. Without a well-rounded digitization phase, HR leaders will not be able to harness the value from HR as well as business data. Additionally, AI adoption requires validated data for training the AI models.

Several new-age companies have a strong inhouse expertise through digital subject matter experts. External HR partners also play a significant role in providing specialized HR services. LinkedIn, a leading professional platform (Linkedin, 2023), mentions that “outside-in” is a way to go as far as designing a new generation HR ecosystem is concerned. The blog mentions that major battlegrounds often lie outside the four walls of HR, such as talent wars. Rather than reinventing the wheel, CHROs can look beyond their organizational boundaries and help their partners adopt AI for win-win relationships that would help create superior business value.

In the wake of AI taking over several processes and jobs, employees in today’s digital age have heightened anxiety due to a fear of deskilling. Organizations need to voluntarily embrace ethical guidelines as part of an overall AI ethics framework so that HR leadership does not cross the line. Resseguier and Rodrigues (2020) advocate that AI adoption can prove counterproductive with negative consequences if due consideration is not given to well-structured AI ethics. Hagendorff (2020) is critical of the way business world has looked at AI ethics. The author has a view that today’s business organizations are not paying attention to ethical guidelines first and then reinforcing them effectively.

AI provides key insights to HR leaders but at the end of the day, it is up to HR leaders to accept, reject, or modify what has been recommended by AI.

## Contributions to theory, practice, and digital policy formulation

**Figure 4:**
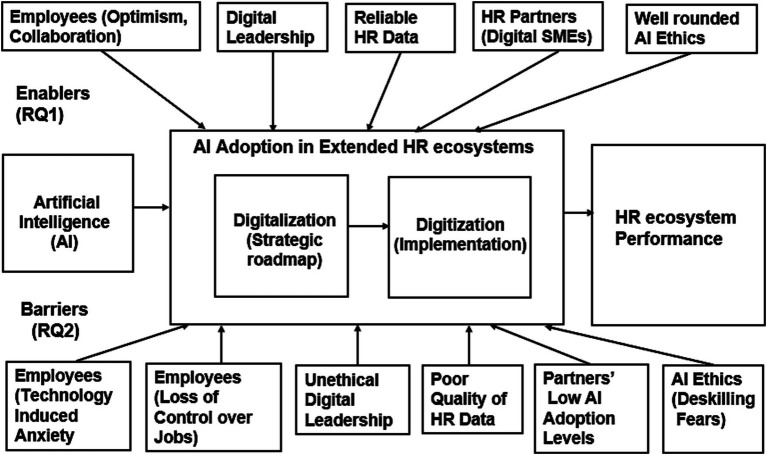
Proposed theoretical model for AI adoption in HR ecosystems.

There is a growing interest among academic researchers to explore the AI adoption issues in HRM. The current academic literature lacks the influence of digital transformation strategy (Verhoef et al., 2021) and AI adoption from a HR performance point of view (Fenech et al., 2019; Tambe et al., 2019). The areas related to the digital transformation of HR and resultant AI adoption are still under-researched. This study contributes to the theory by providing a theoretical model (Figure 4) of AI adoption in an extended HR ecosystem.

As HR strategies are tightly coupled with business strategies, HR performance has a significant impact on overall organizational performance. The research findings are in line with the literature, especially recent articles stating that digital transformation concerns technology as well as factors related to people, processes, and culture, etc. (Trenerry et al., 2021). The proposed theoretical model (Figure 4) addresses the research gap by identifying HRM-specific factors (enablers and barriers), mapped to the research questions (RQ) in extended HR ecosystems. This theoretical model adds to the AI-HRM literature that is a nascent and fast-growing field in the HRM, organizational psychology, and social sciences domains.

**Figure 5:**
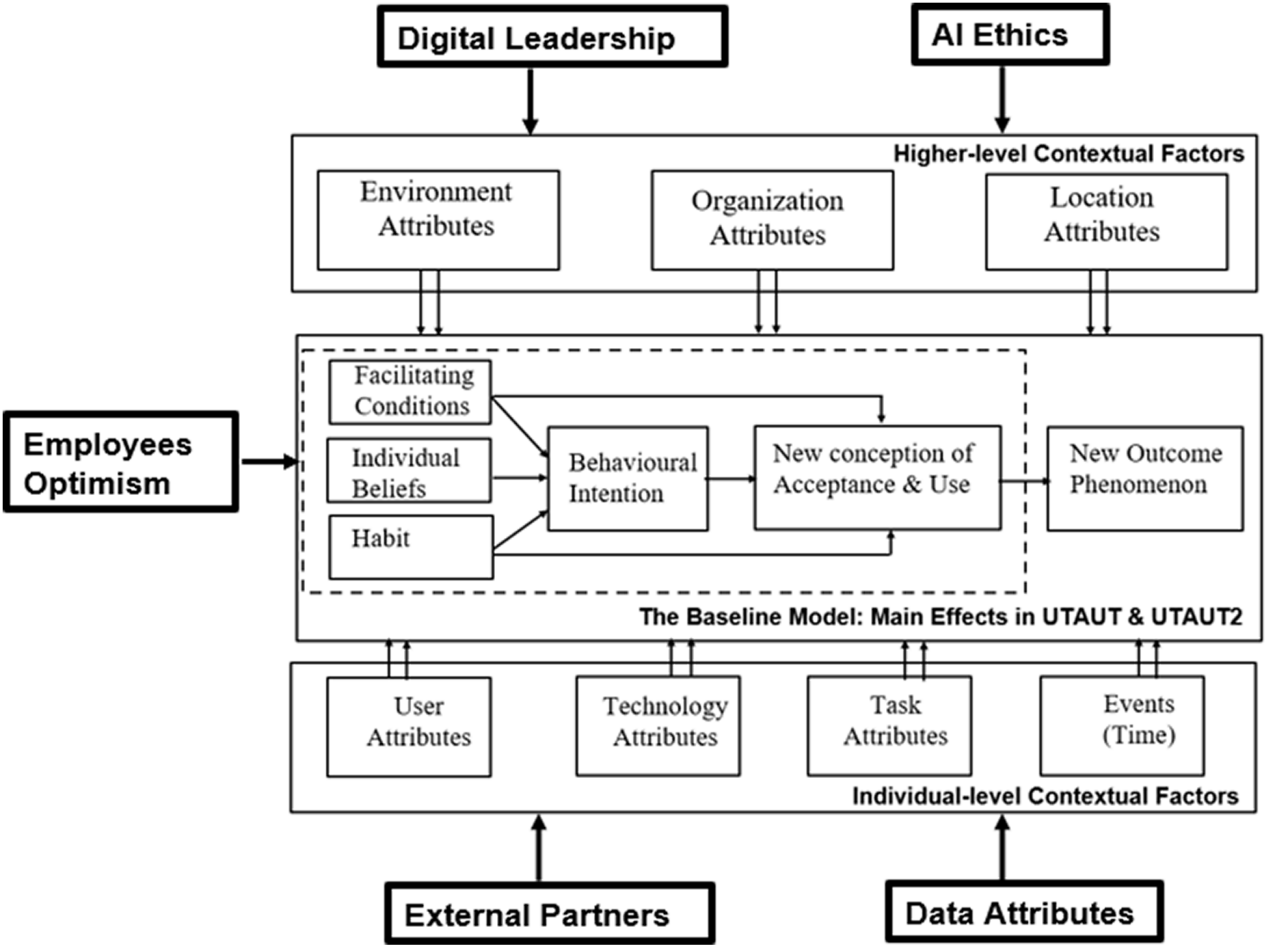
Proposed additions to the UTAUT framework.

This study also proposes additions to the unified theory of technology acceptance and use of technology framework (Venkatesh et al., 2016) as per Figure 5, proposing the addition of new HRM factors in the various layers of the UTAUT multi-layered framework.

Digital leadership is a key organizational attribute as regards the adoption of AI technology in HR ecosystems. In addition, embracing AI ethics also relates to organizational attributes represented as higher-level contextual factors in the UTAUT model (Venkatesh et al., 2016). We have added the optimism of employees as integral to behavioral intention that as per the UTAUT model leads to new outcome phenomena (by way of technology adoption). External partners and data attributes (HR data in the context of this study) are also significant in relation to individual level contextual factors as per the UTAUT model by Venkatesh et al. (2016).

The HR landscape is changing rapidly because of recent AI disruptions. This study provides significant insights to CEOs, CHROs, Chief Information Officers (CIOs), Chief Digital Officers (CDOs), Human Resources Centre of Excellence (HR COE) heads, and digital SMEs regarding the adoption of AI in HRM through best-in-class HR practices. Further, digital natives are set to play a key role in workplaces, so CHROs must leverage their optimism by building a climate of trust and get their whole-hearted support to take the digital transformation agenda forward. This is only possible if there are digital leaders who set a digital tone in HR ecosystems.

HR is an internal customer of a chief digital officer (CDO). This study stresses the need for strong collaboration between HR employees and digital subject matter experts. HR employees need to be up to date with rapid technological changes occurring in the field of AI. The research advises CHROs to focus on upskilling HR employees with adequate digital skills, particularly those who will be part of HR digital transformation projects.

The research cautions HR leaders that excessive dependence on technology could prove counterproductive, as employees experience a lack of a human touch. This study advises CEOs and CHROs to draw a code of AI ethics that can be self-regulated to minimize the technology-induced anxiety, provide a human touch, and remove fears of deskilling from the minds of employees. Leaders must thoroughly address employees’ privacy concerns. The ethical approach will help cultivate a culture of harmonious human–machine collaboration. Malik et al. (2023), in the context of future research directions, advocate that to achieve sustained success, organizations must address the ethical dilemmas in adopting AI technologies (p. 12) and formulate appropriate strategies. The deployment of AI should not be at the expense of employees. Thus, AI ethics would steer the actions of HR leaders in a direction that serves the interests of all stakeholders of the organization, including society.

This study also provides insights for digital policymakers in the public policy domain, such as building strong human capital, creating ecosystems with high learning agility, digital workstyles, upskilling in digital skills, accelerating digital innovation, and future readiness. These insights can be used by policymakers for the formulation of desired policies to support the rapid digitalization in industrial ecosystems.

## Research limitations and future directions

This research focuses on three well-known companies in India and is not set up in a global context. The adoption of AI in many geographies due to cultural differences could offer difficult challenges for HR leaders. Overemphasis on AI has the risk of creating an imbalance, leading to negative organizational and societal outcomes. There is a growing misconception among practitioners that AI can replace humans and, as a result, several jobs can be automated. This has created fear among employees that their jobs are at risk. Budhwar et al. (2022) advocate that on the one hand, AI in HRM leads to positive outcomes but on the other hand, there can be potential negative consequences for an organization and its employees. Rampersad (2020) cautions HR leaders (in the context of robotic process automation) that transitions related to the acquisition of new digital skills must be handled well; with failure to do so, there is a huge risk of massive deskilling as robots replace humans. The issue of algorithmic control in the context of changing the landscape of work also needs to be examined (Joyce et al., 2023).

Cortelazzo et al. (2019) advocate that the role of culture in the selection and implementation of digital technologies needs to be researched. It is a circular issue: digital strategy creates a culture that influences digital strategy. Cortelazzo et al. also mention that digital transformation is not about technology alone; it is the transformation brought in by both people and technology. The impact of business and HR strategies on digital transformation requires further research. There seems to be a significant research gap in studying AI adoption in the context of the fear surrounding its use, including its deskilling aspects. This study focuses on the augmentation of human abilities with AI. This area can be further investigated. Eijnatten and Putnik (2004) point to a concept of dynamically networked enterprises. This research did not study the linkages between different enterprises.

The recent emergence of generative AI as a technology for higher value creation in HR ecosystems is an interesting area of research: “Generative AI has been severely unexplored” (Dwivedi et al., 2023, p. 5). The authors further state that due to the emergence of generative AI technologies, “jobs will be drastically different” (p. 4). Generative AI and large language model (LLM) applications can be easily adopted by companies (Daugherty, 2023). HR leaders can leverage LLM applications for statutory compliances, job descriptions, contract formulation (with employees and partners), etc. Generative AI in extended HR ecosystems is an exciting area of research. This study did not explore these recent advancements.

This study has done a detailed cross-case analysis with respect to three Indian companies (well-known brands) that are at different stages of AI adoption in their respective extended HR ecosystems. The cross-company matrix indicates the mean values of code frequencies and a label in terms of the strength of each theme (e.g., strong, moderate, and weak). In addition, code frequencies (mean values) have been also used for case analysis with respect to digital natives and digital immigrants covered in the study. Future research initiatives can focus upon doing detailed quantitative analysis to establish statistical relationships between the key themes (variables) highlighted in the research findings of this study.

## Conclusion

The HR functions today are on the cusp of a major wave of digital transformation owing to the emergence of artificial intelligence. AI adoption in HRM offers many interesting use cases for human resource professionals that can significantly enhance HR performance. The CHROs and the entire HR team must be ready to embrace the adoption of AI with open arms while keeping AI ethics at the center stage. This study investigated various HRM-specific factors (enablers and barriers) that accelerate or impede the adoption of AI in the extended HR ecosystem (including internal and external partners). AI adoption in the HRM domain is a complex phenomenon as HR leaders deal with sensitive issues related to humans, which can lead to significant organizational and societal concerns. In addition, the HR domain does not generate massive amounts of data that AI algorithms can crunch with ease and provide key insights to HR leaders. Research related to AI adoption in the HRM function is still at a nascent stage and many aspects of AI adoption in extended HR ecosystems are still under-researched. This study aims to fill this significant research gap and contribute to reimagining workplaces, where humans and machines augment capabilities in a harmonious way to enhance the performance of HR ecosystems.

## Data availability statement

The original contributions presented in the study are included in the article/supplementary material, further inquiries can be directed to the corresponding author.

## Ethics statement

The studies involving humans were approved by Institutional Review Board, Indian Institute of Management Indore, IRB approval number: EFPM/28032022/001. The studies were conducted in accordance with the local legislation and institutional requirements. The participants provided their written informed consent to participate in this study. Written informed consent was obtained from the individual(s) for the publication of any potentially identifiable images or data included in this article.

## Author contributions

AS: Conceptualization, Data curation, Formal analysis, Investigation, Methodology, Project administration, Resources, Supervision, Validation, Visualization, Writing – original draft, Writing – review & editing. JP: Conceptualization, Data curation, Formal analysis, Methodology, Validation, Visualization, Writing – original draft, Writing – review & editing.
